# Updating PET/CT performance standards and PET/CT interpretation criteria should go hand in hand

**DOI:** 10.1186/s13550-019-0565-y

**Published:** 2019-10-29

**Authors:** Ronald Boellaard, Terez Sera, Andres Kaalep, Otto S. Hoekstra, Sally F. Barrington, Josée M. Zijlstra

**Affiliations:** 1 Department of Radiology and Nuclear Medicine, Amsterdam University Medical Centre, VUMC, de Boelelaan 1117, 1081 HV Amsterdam, The Netherlands; 2 Department of Nuclear Medicine and Molecular Imaging, University Medical Center Groningen, University of Groningen, Groningen, The Netherlands; 3 EANM Research Limited (EARL), Vienna, Austria; 4 Department of Nuclear Medicine, University of Szeged, Korányi fasor 6, Szeged, Hungary; 5 Department of Medical Technology, North Estonia Medical Centre Foundation, 19 J. Sütiste Street, Tallinn, Estonia; 6 King’s College London and Guy’s and St Thomas’ PET Centre, School of Biomedical Engineering and Imaging Sciences, King’s College London, Amsterdam, UK; 7 Department of Hematology, Cancer Centre Amsterdam, Amsterdam UMC, Vrije Universiteit Amsterdam, de Boelelaan 1117, 1081 HV Amsterdam, The Netherlands

## Abstract

This letter aims at explaining that adjusting the performance of PET/CT systems to a new standard also requires updating of interpretation criteria. Simply changing one aspect of the imaging procedure, i.e., PET/CT performance and image quality, and not adapting interpretation criteria will result in an increase of false positive (or negative) reads.

## Correspondence

Dear editor,

Ly et al. recently published a paper on the impact of the newly proposed EARL standards on Deauville scores in lymphoma 18F-FDG-PET/CT studies compared with current EARL standards [1]. We would like to compliment the authors with this clear paper, which again emphasizes the need to apply interpretation criteria in a validated manner considering the underlying image quality and imaging procedures. In particular, moving to new PET/CT performance standards should never be implemented clinically without properly evaluating its implications for visual reads and/or quantitative evaluation criteria. The authors have nicely demonstrated this in case of Deauville scoring of lymphoma 18F-FDG-PET/CT studies. We also would like to point out that many sites already adopted new PET reconstruction technologies that alter visual and quantitative reads substantially, apparently without awareness of the implications or impact on image interpretation [2].

The newly proposed EARL criteria were developed to allow use of new PET/CT technologies, such as time of flight, digital PET detectors, and so-called point spread function reconstructions [3]. This update was required to avoid that EARL standards would become prohibitive to improve image quality and to benefit from new PET technology improvements. These new EARL standards result in improved contrast recovery and lesion detectability, which was the main intention for exploring the feasibility of a new standard. Specifically, smaller lesions (< 1.5 cm diameter) will become (more) visible and will show substantially increased SUVs compared to that seen with current standards, as was also observed by Ly et al. [1]*.*

Moving to a new standard should thus never be done without care. There are several strategies to accommodate this transition. First, both for visual and quantitative reads, the PET images can be downgraded by a simple filtering step to become compliant to the current EARL standard. This can be done explicitly by performing a second reconstruction or simply applying a Gaussian filter to convert images from new EARL to current EARL compliance [4]. In these cases, a second dataset is generated with a lower spatial resolution, which may not always be desired by clinicians and readers. In order to facilitate moving to the new EARL standards, during the following 2 years, EARL will harmonize the scanners for both set of standards while the application of the new standard can be implemented in clinical practice.

A similar approach is the so-called EQ.PET method which is commercially available on specific systems and extensively explored by, e.g., Lasnon et al. [5]. This method actually downgrades the images as well, but only during quantitative reads. This filtering is performed in the background of the PET/CT vendor-provided software, such that the reader is still presented with a high spatial resolution image. A possible downside of this approach is that EARL-compliant images are not generated. This could result in incorrect reads when PET images are evaluated using other display stations or software. Another strategy would be to adapt the current interpretation criteria to the new standards [6]. In order to achieve this, similar studies as carried out by Ly et al. [1] can be performed in which visual and quantitative reads are produced on the same data but with two or more reconstructions that can be directly compared. In this way, new criteria can be derived and cross-calibrated against current ones.

The best way to move forward will become clear in the future when more studies such as the one by Ly et al. have been performed. However, it is inevitable that both PET/CT performance standards, such as EARL, as well as visual and quantitative interpretation criteria need to evolve along with the evolution of PET technologies. Readers should be aware that mismatches between these standards and image evaluation criteria will result in incorrect PET/CT reads [2], not only for lymphoma studies. The paper of Ly et al. [1] is another clear example showing the need for our community to pay careful attention to PET/CT harmonization and to apply interpretation criteria with caution, being aware that these criteria are only valid in combination with “matched” PET/CT performance standards.

## Data Availability

Not applicable
